# Plasticity of vibratory courtship in response to nuptial gift quality in male *Pisaura mirabilis*

**DOI:** 10.1093/beheco/arag053

**Published:** 2026-05-14

**Authors:** Morgan M Oberweiser, Mariève E Hébert, Monika J B Eberhard

**Affiliations:** Department of General and Systematic Zoology, Zoological Institute and Museum, University of Greifswald, Loitzer Str. 26, Greifswald 17489, Germany; Department of General and Systematic Zoology, Zoological Institute and Museum, University of Greifswald, Loitzer Str. 26, Greifswald 17489, Germany; Department of Biology, Institute of Cell and Systems Biology of Animals, University of Hamburg, Martin-Luther-King-Platz 3, Hamburg 20146, Germany

**Keywords:** Biotremology, multimodal courtship, investment, nuptial gifts, trait plasticity

## Abstract

Courtship is often a multimodal process involving several signals of different modalities occurring simultaneously. Male nursery web spiders (*Pisaura mirabilis*) perform substrate-borne vibrations while offering a nuptial gift (a nutritional donation which also serves as a visual and olfactory signal) as they court females. The nuptial gift, consisting of a prey item wrapped in silk, is a considerable investment for the male, and vibration is also likely an energetically costly signal. In this study, we investigate how these 2 expensive mate attraction tactics interact in order to explore how investment might be partitioned between them. We conducted behavioral experiments where male *P. mirabilis* were provided with nuptial gifts in 3 treatments—no gift, medium gift, large gift—and recorded their courtship vibrations in repeated trials (once with each nuptial gift treatment). We found that calling duration (the total amount of time spent signaling) is longer when males have a nuptial gift vs. when they do not, but the duration is not plastic in response to the mass of the gift. Pulse rate is not affected by the quality of the nuptial gift. In trials where a gift is present, only pulse interval consistency changes based on gift quality, with intervals becoming more consistent (putatively more attractive) as gifts increase in mass. Our results suggest that male spiders do adjust their investment in vibratory courtship signals at a general level, though the plasticity is slight.

## Teaser

Courtship of male *Pisaura mirabilis* spiders includes two costly tactics: a vibratory signal and a nuptial gift. We asked whether males adjust their vibratory performance based on gift quality (mass). In experiments where males had gifts of different sizes, they signaled longer when they had any gift, but signal features changed only slightly with gift size. These results suggest that males do adjust their courtship signal in response to the presence of a nuptial gift, but less so in response to the size of the gift.

## Introduction

Secondary sexual characteristics such as weapons, ornaments, and courtship behavior usually provide higher reproductive success despite being potentially detrimental to survival (Zahavi 1975; Jennions et al. 2001). Such traits, most often described in males, are typically costly (eg, in terms of development, energy expenditure, conspicuousness toward predators) and condition-dependent (Simmons et al. 2017). It is expected that investment in secondary sexual traits is flexible so that individuals can accommodate the high cost of expressing them; without plasticity in resource allocation, these traits cannot arise or be maintained under sexual selection (Jennions et al. 2001).

When multiple secondary sexual traits are expressed at once, complicated relationships can arise regarding the partitioning of resources and/or effort between traits (Jennions et al. 2001). Within a multimodal display of courtship, where at least 2 sensory modalities are utilized concurrently, different sexually selected traits combine, interact, and create a signal that becomes more than the sum of its parts (Mitoyen et al. 2019). When several different signaling systems are in play, males might show plasticity in courtship tactics based on environmental conditions or available resources (Bro-Jørgensen 2010). For example, some wolf spiders that usually court with both visual and vibratory signaling will adjust their display depending on their signaling environment, using more visual signals on substrates that transmit vibrations poorly (Gordon and Uetz 2011). While trait interaction within multimodal displays is a subject of growing interest, still more empirical and experimental studies are needed to uncover how these complicated signals function and evolve (Mitoyen et al. 2019).

*Pisaura mirabilis*, the European nursery web spider, has a multimodal courtship display involving substrate-borne vibrations and a nuptial gift. Commonly seen in arthropods, a nuptial gift is a material donation transferred from male to female during copulation, which can enhance reproductive success even though its production incurs nutritional and/or energetic costs to the male (Gwynne 2008). In the case of *P. mirabilis*, sexually mature males produce gifts by wrapping caught prey in silk, and carry them in their chelicerae while seeking out mating opportunities (Nitzsche 2011). It is difficult to classify the nuptial gift of *P. mirabilis* under any single signal modality, because it is essentially a valuable food resource for the female (Austad and Thornhill 1986), but additionally functions as a visual (Stålhandske 2002; Prokop and Provazník 2025) and olfactory signal (via silk-borne chemicals) (Beyer et al. 2021). Upon contact with female dragline silk, a male shows sexual excitement in the form of opisthosoma tremulations, whole-body jerks, and leg rubbing (Lang 1996; Nitzsche 2011; Eberhard et al. 2020). The tremulation of the opisthosoma generates a substrate-borne vibration made up of short regularly-repeated pulses, which is characteristic to *P. mirabilis* courtship and thought to be a potential signal of male quality (Oberweiser et al. 2025).

Nuptial gifts in *P. mirabilis* have been well-studied in the past decades. Males that present a gift are more likely to be accepted for mating than males that do not (Stålhandske 2001; Prokop and Maxwell 2009; Maxwell and Prokop 2018). It is known that males with larger nuptial gifts obtain longer copulations (Bruun et al. 2003), and that a longer copulation results in more eggs being fertilized (Stålhandske 2001). Despite these advantages, carrying a nuptial gift does incur costs such as reduced running speed (Prokop and Maxwell 2012) and higher metabolism (Prokop and Okrouhlík 2021). Males also invest into the silk that they use to wrap the gift, to the benefit that females take longer to digest nuptial gifts with more silk, leading to longer copulations (Lang 1996). Gift wrapping is condition-dependent, as satiated males spend more time wrapping and use more silk than starved males (Albo et al. 2011a, 2011b), and time spent wrapping the gift is commonly used as a proxy for male investment in courtship (Tuni et al. 2017). Effort in gift production is shown to be plastic in a handful of scenarios: males reduce silk investment (but increase speed of gift production) when they perceive a high risk of intrasexual competition (Tuni et al. 2017), they increase silk investment when in contact with draglines of adult and hungry (versus sub-adult and satiated) females (Ježová et al. 2023), and males invest more effort in gift production in darker environments, presumably to increase the visibility of the gift (Prokop and Provazník 2025).

Much less is known about the courtship vibrations produced by males when they contact female dragline silk. Recent research has shown that the vibratory courtship signals are condition-dependent: starved males show a longer latency to start calling and emit fewer pulses than males in good condition (Eberhard et al. 2020). Females are more likely to accept males in good condition than starved males, suggesting that this courtship behavior might serve as a signal of male quality (Eberhard et al. 2020). Specifically, the vibratory traits of calling duration and pulse rate have been identified as potential signals for female choice as they are highly consistent within individuals (though, pulse interval consistency was found to have high within-individual variability) (Oberweiser et al. 2025). Heavier males signal for a longer duration of time, and younger males signal at a faster rate, further suggesting that these traits might correlate with the physical quality of the male (Oberweiser et al. 2025). Altogether these aspects of vibratory courtship (condition-dependence, correlation with quality traits, within-individual consistency) indicate by association that the signal would be well-suited as an indicator of male quality and a criterion in mate selection (Jennions et al. 2001; Schuett et al. 2010), though as of yet there is no direct evidence of female behavior being affected by this signaling modality (ter Haar et al. 2025). Interestingly, while information about the vibratory signal is relatively scarce, there is evidence of this signal being adaptable based on female sexual maturity: males that encounter sub-adult female silk are less likely to signal, and produce fewer pulses when they do signal (Eberhard et al. 2021). While the courtship tremulation signal of *P. mirabilis* has gained recent interest with these few studies, the relationship between the nuptial gift and vibratory courtship signal has not yet been investigated.

The aim of this study is to examine the impact of nuptial gift quality on the vibratory signaling behavior of *P. mirabilis* males by subjecting them to 3 treatments in the presence of female dragline silk: no nuptial gift (N), medium-sized nuptial gift (M), and large-sized nuptial gift (L). We propose that *P. mirabilis* males take an “all eggs in one basket” approach, and invest heavily in vibratory courtship when they are afforded the resources to build a higher-quality gift. As the males cannot rely on the physical characteristics of the gift such as color, shape, or size to influence female acceptance rate (Andersen et al. 2008; Albo et al. 2012; Maxwell and Prokop 2018), they may adjust the intensity of vibratory courtship to enhance their overall multimodal courtship display so that their high-investment nuptial gift is utilized before degrading or desiccating. We therefore hypothesize that males with heavier nuptial gifts engage in more costly vibratory courtship. Such a correlation would be an example of honest signaling; it should be noted that while dishonest signaling does occur in *P. mirabilis* via worthless gifts (Albo et al. 2011a, 2011b; Ghislandi et al. 2014), it was not considered in this experiment since male spiders were always provided with nutritious material for the building of their nuptial gifts. Costlier signaling is presumed to be attractive to females, as only good-quality males should have the resources to invest in expensive courtship (given that the signals are honest) (Jennions et al. 2001). Therefore, we expect that males with heavier nuptial gifts will have longer calling durations, higher pulse rates, and more consistent pulse intervals (measured as smaller coefficient of variation).

## Methods

### Spider collection and rearing

We collected male and female sub-adult *P. mirabilis* spiders around Greifswald, Germany (54° 05 ′ 38.1′′ N 13° 22′ 17.9′′ E) between March and May of 2024. The spiders were housed inside individual plastic vials, one end covered with mesh, the other sealed with a sponge standing in water in order to maintain high humidity. Each vial contained a plastic plant. We kept the spiders under alternated full-spectrum and UV lamps (ExoTerra Natural Light, ExoTerra Reptile UVB100, ExoTerra Repti-Glo UV) from 9:00 AM to 7:00 PM at approximately 24 °C and sprayed them with water daily. We fed them 1 fly (*Lucilia caesar*) twice per week. Feeding was withheld for 3 days before the first trial to ensure the spiders interacted with the flies provided for nuptial gift production.

### Experimental setup

We constructed an arena for the collection of vibratory data by stretching and fixing a 13 cm diameter circle of 80 denier nylon fabric over the 20 cm opening of a plastic cylindrical pipe. We placed the arena in a tub containing sand in order to dampen external vibrations. A Polytec PDV-100 portable laser Doppler vibrometer (Polytec Gmbh, Waldbronn, Germany) was used to measure vibratory signals, with the laser directed at a small piece of reflective tape (ca. 0.5 cm^2^) affixed directly to the fabric in the center of the arena. The laser was operated with a velocity of 20 mm/s, with a low-pass filter of 22 Hz. We used black cardstock to create walls around the arena, preventing the spiders from escaping and reducing disruption. The measurements from the laser were digitized (USB-connection-box VIB-E-220, Polytec) and stored using the Software VibSoft 5.3 (Polytec Gmbh, Waldbronn, Germany).

### Experimental procedure

We subjected 39 males, aged 7 to 21 days after their final molt, to 3 experimental treatments in random order: no nuptial gift (N), a medium-sized nuptial gift (M) and a large nuptial gift (L). For the M treatments, we provided males with *Lucilia caesar* flies (body length 5.5 to 11.5 mm) to produce their nuptial gifts, while *Calliphora vomitoria* flies (body length 9 to 14 mm) were used for the L treatments (Sivell 2021). We placed the fly in the males’ vials in the morning, and allowed them a minimum of 1 h to catch and wrap it with silk. If a male captured a fly but failed to wrap it within several hours, he was still included in the experiment as intended, as females accept males that present an unwrapped gift at a similar rate to those with a wrapped gift (Bilde et al. 2007). If a male of the M or L treatment failed to catch the supplied fly within the experimental period, we removed the fly, and the N treatment was conducted instead, given that the male had not previously completed it. The M or L treatment was reattempted the next day with a new fly. Therefore, 12 males underwent the trials in the “NML” sequence, 9 in the “NLM”, 8 in the “MNL”, 4 in the “MLN”, and 3 each in the “LNM” and “LMN” sequences. While this approach affected the randomness of the order of treatments, it allowed the testing of a greater number of males within the time constraints. Despite the challenges, this experimental design supported a within-subject approach, where we could repeatedly test individuals in order to assess their behavioral plasticity in response to multiple treatments, as well as to increase statistical power and reduce the amount of between-individual variation affecting our analysis (Charness et al. 2012; Dingemanse and Dochtermann 2013). Each male underwent experimentation once per day, over a maximum of 4 days.

**Figure 1:**
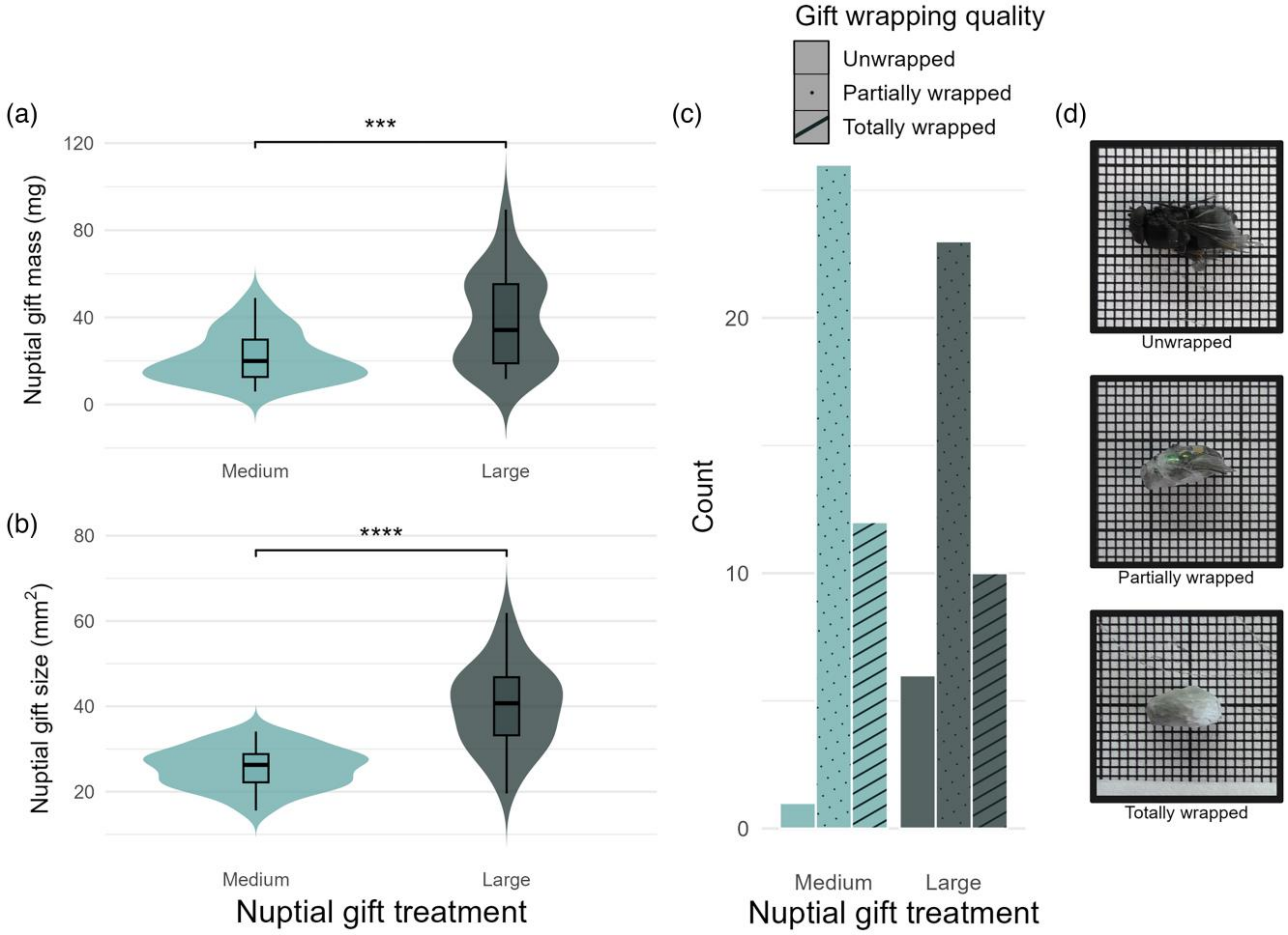
Nuptial gift attributes according to treatment: violin plots describe a) nuptial gift mass and b) nuptial gift size, wherein boxplots indicate the interquartile range with the horizontal line denoting the median, and whiskers indicate 1.5× the interquartile range. Nuptial gift wrapping quality is summarized by c) a bar graph describing the number of each quality type per treatment, as well as d) examples of the levels of nuptial gift wrapping quality, where 1 small square = 1 mm^2^.

We used 4 unmated females in this experiment, aged between 8 and 28 days from their final molt. For each trial, we randomly selected 1 female and placed her in the arena for 5 minutes to deposit dragline silk, which is a known stimulant of male courtship vibration (Scott et al. 2018; Eberhard et al. 2021). We then removed the female, started the laser recording, and immediately placed the male (holding his nuptial gift, if applicable) inside the arena. We recorded vibrations for 350 s, after which we removed the male. We retrieved nuptial gifts using forceps, photographed them on millimeter paper (Panasonic HC-V777 HD camcorder) (Fig. 1d), and weighed them on an analytical scale (KERN ABS-N/ABJ-NM). Using the photos and the program ImageJ (Schneider et al. 2012), we measured gifts along and perpendicular to their longest axis. These measurements were used to estimate the size of the gifts using the formula for the area of an ellipse A=πab, where *a* and *b* are the semi-major and semi-minor axes. We subjectively rated the gifts as unwrapped, partially wrapped, or fully wrapped based on the photographs (Fig. 1d). The arena was cleaned with 70% to 80% ethanol between each trial to remove silk and possible chemical pheromones. After their final trial, we weighed males alive (KERN ABS-N/ABJ-NM), euthanized them in the freezer at −32 °C, and preserved them in ethanol (70% to 80%). We cut both fourth legs at the femur and measured the length of the tibia-patella at a consistent diagonal with a stereomicroscope (ZEISS SteREO Discovery v20 with AxioVision 4.9.1). The average of the 2 measurements was used for all spiders (except for one that was missing a leg) as a proxy for body size (Jakob et al. 1996). We calculated a body condition index (BCI) for each male using the residuals of a linear regression of mass on leg length, after log transformations (Jakob et al. 1996).

### Quantifying vibration

We analyzed the vibratory recordings as .wav files using the pulse train analysis feature of Avisoft sound analysis and synthesis software (Avisoft Bioacoustics, Glienicke/Nordbahn, Germany), resulting in measurement free of observer bias. Vibration was quantified according to Oberweiser et al. (2025), though the filtering of pulse interval measurements was slightly modified. Our dataset was limited to the range of the median +/− the inter-quartile range. This range spanned from 0.237 s to 1.043 s, resulting in a normal distribution (verified by visual assessment of a histogram and Q-Q plot) around the median pulse interval duration of 0.64 s. The resulting summary variables calculated for each trial included calling duration (sum of all pulse interval durations, in seconds) and pulse rate (the number of pulses measured divided by the calling duration, in pulses/second). We additionally calculated the pulse interval coefficient of variation (CV) as a measure of pulse interval consistency for each trial. CV is calculated by dividing the standard deviation by the mean, resulting in a simple indicator of dispersion with a value approaching 0 describing an absence of variability and a value approaching 1 describing high relative variability. While the usefulness of CV has been questioned for in-depth analyses (Reinhold 2011; Eberhard and Treschnak 2018; Pélabon et al. 2020), and has been shown to differ from the results of highly complex models testing within-individual variation in this very same context (pulse interval CV of *P. mirabilis* courtship vibration) (Oberweiser et al. 2025), we judged it to be an appropriate measurement of pulse interval variation in our experiment. We posit that despite high within-individual and within-trial variation of the pulse interval itself, assessing pulse interval CV in relation to nuptial gift quality may offer more insight into whether this trait is utilized as a function of courtship.

### Statistical analysis

We assessed the differences in measured vibratory parameters between nuptial gift treatments using repeated measures ANOVAs and nonparametric Friedman tests, depending on data normality. We used a paired T test with Bonferroni adjusted *P*-value as a post-hoc test. Furthermore, we used general linear mixed models (GLMM) to evaluate the relationship between vibratory variables and nuptial gift mass, using the “glmmTMB” package (Brooks et al. 2017). These models exclude data from the N treatments, as there is no gift to weigh. We employed a backward elimination approach to identify the model that best fit the data, starting with nuptial gift mass, female ID, male BCI, and trial order as factors. Male ID was included as a random effect to account for the repeated measures (female ID was included as a fixed rather than random effect because there were only 4 levels and therefore not enough variation to make it appropriate as a random effect). The Gaussian family was employed for all models. We checked the best-fitting model diagnostics visually using the “DHARMa” package (Hartig 2024), and calculated marginal and pseudo R values using the “MuMIn” package (Bartoń 2025).

## Results

A total of 39 male spiders were tested in 3 consecutive trials. Nuptial gifts in the M treatment had a mean mass of 21.68 mg (SE = 1.80) and mean size of 25.69 mm^2^ (SE = 0.76), and nuptial gifts in the L treatment had a mean mass of 38.67 mg (SE = 3.38) and a mean size of 39.85 mm^2^ (SE = 1.55) (Fig. 1a and b). A previous study with nuptial gifts collected from the wild reports natural gifts to have a mean mass of 9.6 mg (SE = 1.6, *n* = 23) (Prokop and Maxwell 2012), and another reports that around 70% of wild-collected gifts were below 10 mm^3^ in volume when the total range spanned from 2.4 to 42.5 mm^3^ (Nitzsche 1999). Therefore, we believe our sample accurately represents medium and large-sized nuptial gifts, even though gifts of this size are less commonly found in the field. Nuptial gifts were significantly different in both mass and size (estimated by ellipse area using the semi-major and semi-minor axes) between the 2 treatments, with males in the L treatment having both heavier (Wilcoxon test, N = 78, W = 1,137, *P* < 0.001), and larger (Wilcoxon test, N = 78, W = 1,383, *P* < 0.0001) gifts than those in the M treatment. There was, however, overlap between the mass and size ranges of the 2 treatments (Fig. 1a and b). The mass of the nuptial gifts was positively correlated with their size (Pearson correlation, *r*(76) = 0.666, *t* = 7.7805, *P* < 0.0001). In both treatments the majority of gifts were partially wrapped, with more unwrapped gifts occurring in the L treatment than the M treatment (Fig. 1c); this is consistent with the fact that in the field, smaller gifts were more frequently found wrapped than unwrapped (Prokop and Semelbauer 2017).

### Comparison of courtship signals

**Figure 2:**
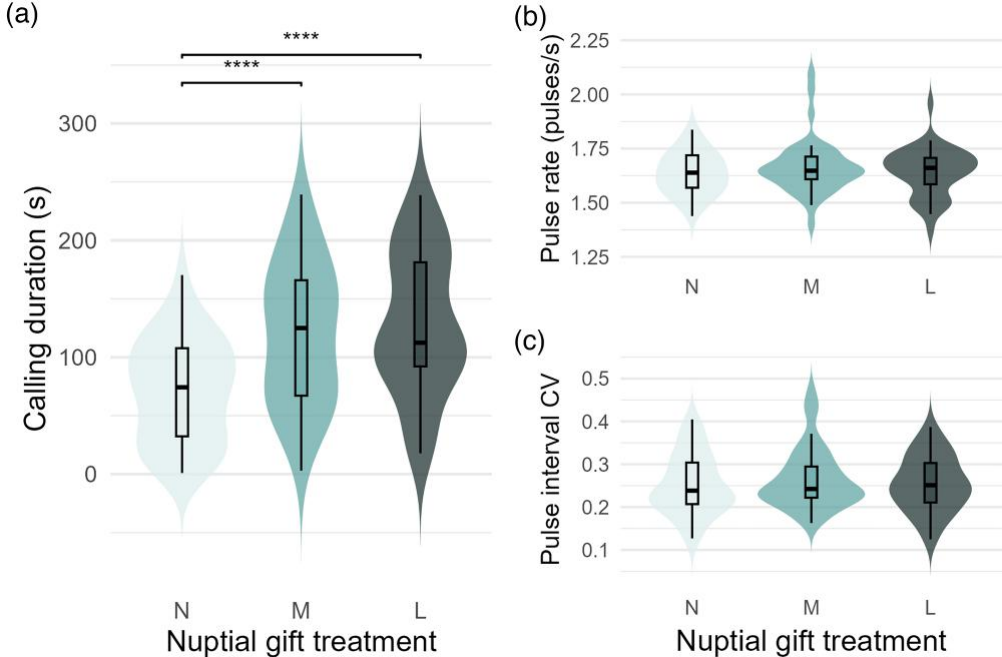
Vibratory parameters as measured between the 3 nuptial gift treatments of No gift (N), Medium gift (M) and Large gift (L). Boxplots indicate the interquartile range with the horizontal line denoting the median, and whiskers indicate 1.5× the interquartile range. a) Calling duration differed significantly between the N treatment and the M and L treatments. b) Pulse rate and c) Pulse interval CV did not significantly differ between nuptial gift treatments.

All males produced courtship signals during the trials. Calling duration varied significantly between treatments (repeated measures ANOVA, F(2,76) = 18.60, *P* < 0.001). Males invested less time in signaling during the N treatment compared with the 2 trials where they did have a nuptial gift, but no difference was found between the M and L treatments (Fig. 2a). There was no significant difference among treatments for the other vibratory variables tested, the pulse rate (Friedman ANOVA, 2(2) = 1.59, *P* = 0.452) and pulse interval CV (Friedman ANOVA, 2(2) = 2.67, *P* = 0.264; Fig. 2b and c).

**Figure 3:**
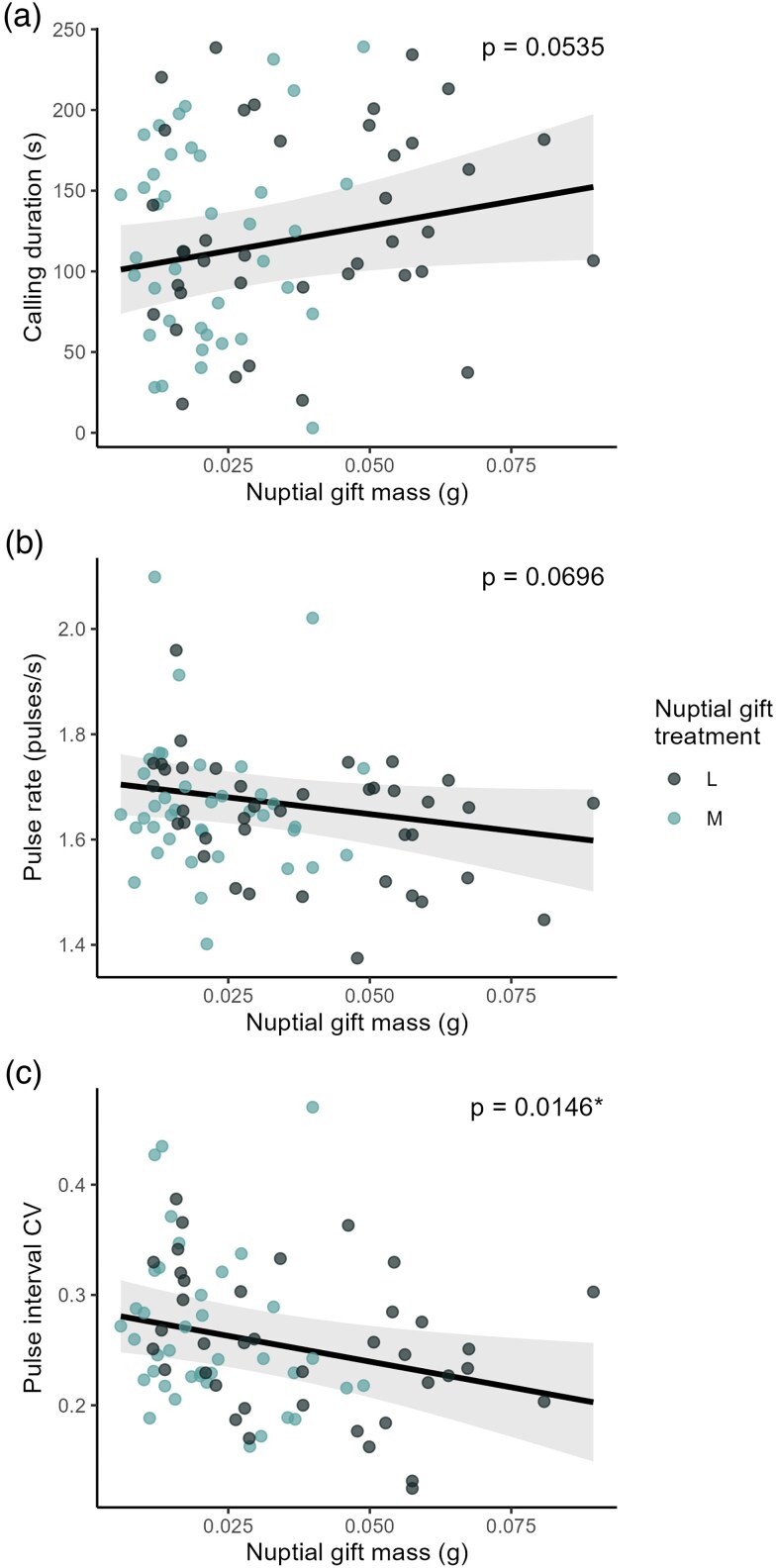
Effect of nuptial gift mass on a) calling duration b) pulse rate and c) pulse interval CV as predicted by the GLMMs. Only pulse interval CV shows significance at *P* < 0.05.

GLMMs were applied to further investigate the relationship between the vibratory parameters and the mass of the nuptial gift (Table 1). Fixed effects of nuptial gift mass, female ID, male BCI, and trial order were initially included. Through the backward elimination method, we determined that male BCI and trial order did not contribute to the models for any of the response variables, so these effects were excluded. Therefore, the best-fitting model in each case included nuptial gift mass and female ID as fixed effects, with male ID as a random effect. None of the fixed effects were found to significantly influence the calling duration or pulse rate. However, nuptial gift mass had a significant influence on pulse interval CV, with heavier nuptial gifts associated with a decrease in pulse interval CV (an increase in consistency; Fig. 3).

**Table 1:** Results of GLMMs with male ID as random effect and nuptial gift mass and female ID as fixed effects.

Response variable	Factor	Χ^2^	df	*P*-value	Marginal *R*^2^/ Conditional *R*^2^
Calling duration (s)	Nuptial gift mass	3.7284	1	0.0535	0.0767/0.5133
	Female ID	5.0589	3	0.1675	
Pulse rate (pulses/s)	Nuptial gift mass	3.2923	1	0.0696	0.0806/0.2795
	Female ID	2.4797	3	0.4790	
Pulse interval CV	Nuptial gift mass	5.9626	1	0.0146	0.0675/0.3608
	Female ID	0.6137	3	0.8933	

Results are reported from Type III Wald chi-squared tests, as well as marginal and conditional pseudo *R*^2^ values. Note that the N treatment (no nuptial gift) was excluded from this part of the analysis since there is no nuptial gift to measure.

## Discussion

Our prediction that *P. mirabilis* males would produce more attractive (ie, more costly) courtship signals in response to the quality of their nuptial gift is partially supported. Males holding a nuptial gift did signal for longer than males without a gift, though there was no plasticity in calling duration based on nuptial gift size. Neither pulse rate nor pulse interval CV (measuring pulse interval consistency) was affected by the categorical nuptial gift treatments. However, within the trials which included a nuptial gift, the mass of the gift itself affected pulse interval CV, with heavier nuptial gifts associated with more consistent (putatively more attractive) pulse intervals.

Calling duration, or the time spent actively signaling during the behavioral trial, indicates a clear investment on the part of the male spider (Seymour and Sozou 2009). Not only is vibratory signaling thought to be highly energetically costly (in comparable Lycosid spiders, drumming displays increased male metabolic rate by a factor of 22 [Kotiaho et al. 1998]), but as with any courtship display, the longer a male spends actively signaling, the less time and energy he will have for foraging, and the more exposed he will be to predation. Our results support the conjecture that in the case of calling duration, males will choose a tactic of simultaneous investment into both the nuptial gift and vibratory signal. However, the calling duration was not shown to be plastic to match the degree of display investment with the quality (mass) of the nuptial gift. Only the presence of a nuptial gift, regardless of mass, caused the males to invest more into courtship by spending more time actively signaling. In those trials with no nuptial gift, the decrease in calling duration could potentially be attributed to a longer latency to initiate vibratory courtship, more pauses in signaling, or stopping signaling prematurely. It has already been found in *P. mirabilis* that starved males and males in contact with sub-adult female silk show a greater latency to signal, suggesting that a longer latency reflects low investment in courtship (Eberhard et al. 2020, 2021; Ježová et al. 2023). This is consistent with our finding that a male in a sub-optimal courtship scenario (ie, lacking a nuptial gift) would invest less in courtship and retain resources for a better chance at securing copulation in the future. Regardless of patterns of male investment, calling duration of the vibratory courtship signal has not yet been directly linked to mating likelihood in *P. mirabilis.*

Pulse rate did not change across the 3 nuptial gift treatments, and was not affected by the mass of the nuptial gift. This fails to meet our hypothesis that males would perform higher-quality vibratory signals when they possess a higher-quality gift. One explanation for this result could be the physical toll of handling and carrying a larger and heavier nuptial gift. Previous studies show that both running speed (Prokop and Maxwell 2012) and metabolic rate (Prokop and Okrouhlík 2021) are negatively impacted by the possession of a nuptial gift (though, fighting success and mobility are not affected [Prokop and Maxwell 2012; Prokop 2019]). Given that males show a distinct preference for building larger gifts (Prokop 2019), and that larger gifts secure longer and more successful copulations (Bruun et al. 2003; Maxwell and Prokop 2018), it is likely that male spiders simply prioritize the size of the nuptial gift over their display quality in terms of pulse rate. Additionally, it has never been directly shown that *P. mirabilis* females have a preference for higher pulse rates, and female preference for pulse rate varies between spider species. In the wolf spider *Hygrolycosa rubrofasciata*, males display consistent pulse rates with little variation between individuals, meaning that this trait is not likely to be subjected to female choice (Rivero et al. 2000). Conversely, in the orb-weaver *Argiope keyserlingi*, females take longer to approach males that shudder at a faster rate, because these males are not able to maintain a long signal duration (Wignall and Herberstein 2013). Thus, while a high pulse rate can be an indication of male quality due to its high energy cost, it may not necessarily be a factor in female choice, and might even negatively impact other aspects of vibratory courtship which may be prioritized by females.

Pulse interval consistency is the only variable which showed plasticity in the nuptial gift treatments, with pulse intervals becoming more consistent as nuptial gift masses increased. Typically, more consistent vibratory signals are thought to be indicators of higher quality and attractiveness, as shown in katydids (De Luca and Morris 1998) and bromeliad spiders (Schüch and Barth 1990). However, preference surrounding pulse interval consistency is not known for *P. mirabilis* females, and in fact, recent analyses of vibratory trait variation in this species indicate that pulse interval consistency is highly variable within individuals, and is likely not a trait important to female choice (Oberweiser et al. 2025). It is speculated to more likely have a function in species recognition, as is the case in stink bugs (Miklas et al. 2003), psylloids (Liao and Yang 2015), and *Cupiennius* spiders (Barth and Schmitt 1991). Furthermore, pulse interval consistency does not seem to be condition-dependent in *P. mirabilis*, as a previous study showed no significant difference in within-male variability between well- and poor-fed males (Eberhard et al. 2020). Therefore, while the fact that *P. mirabilis* males produce more consistent pulses with heavier nuptial gifts aligns with the hypothesis that they generate more attractive signals when they have higher quality nuptial gifts, we treat this result with caution given the contradictory evidence that pulse interval consistency is likely not a trait important to mate selection.

Further research on the criteria of female choice in *P. mirabilis* is essential in order to understand which courtship signals are most attractive, considering not only mate acceptance but also copulation duration, which is positively correlated with the amount of sperm transferred and the number of eggs fertilized (Stålhandske 2001; Albo et al. 2013). The vast majority of current research indicates that the most important factor to directly affect female choice is the nuptial gift; its presence positively influences female acceptance (Stålhandske 2001; Maxwell and Prokop 2018), with female hunger level (Prokop and Maxwell 2009) and the silk-borne chemicals on the gift (Beyer et al. 2021) shown to contribute as well. The presence of the nuptial gift even influences cryptic female choice, with females storing more sperm from males with nuptial gifts than those without (Albo et al. 2013). However, females demonstrably ignore physical characteristics of the nuptial gift such as color, shape, and size, possibly due to the fact that dishonest signaling is seen to occur in this species (Andersen et al. 2008; Albo et al. 2012; Ghislandi et al. 2014; Maxwell and Prokop 2018). While dishonest signaling via worthless gifts (constructed of dry insect exuviae or even plant material wrapped in silk) is a known alternative reproductive strategy, it is fairly uncommon and unstable, and is most likely driven by irregular and seasonal factors such as high male-male competition and low prey availability (Ghislandi et al. 2014). Despite the fact that honest signaling is typically the more reliable courtship strategy, Ghislandi et al. do speculate that deceptive gift-giving behavior might prompt females to preferentially evaluate courtship performance factors such as vibrations rather than nuptial gift traits (2014). The vibratory courtship signal, though, has only indirect evidence so far to link it to female choice: males in better body condition both performed higher-quality courtship vibrations and had higher mating success than starved males (Eberhard et al. 2020). The signal is characteristic, condition-dependent, and contains several individually-consistent components (Nitzsche 2011; Eberhard et al. 2020; Oberweiser et al. 2025), so it seems likely to work in combination with the well-established nuptial gift in a reproductive context. Our results suggest that the actual production of vibratory courtship has some association with female choice, as the presence of the nuptial gift affected calling duration. However, the fine-scale characteristics of the signal (pulse rate, pulse interval consistency) may not be as important for female assessment of male quality, as the correlation of these traits with nuptial gift quality was either absent or less pronounced.

Courtship in *P. mirabilis*, like in the majority of animal systems, is a multimodal process; it involves multiple signals of different modalities being transmitted concurrently, and potentially transmits multiple pieces of information from the male to the female. Besides the nuptial gift and vibratory signal discussed in this study, *P. mirabilis* courtship also includes a chemical component, and visual/motion-based signals such as leg waving/rubbing (Nitzsche 2011; Beyer et al. 2021). The nuptial gift itself constitutes such a complicated factor because it is not a “signal” per se, but rather a collection of signals (visual, chemical) plus a nutritional resource which has a direct effect on female fitness and fecundity (Austad and Thornhill 1986; Stålhandske 2002; Beyer et al. 2021; Prokop and Provazník 2025) (though, the direct benefit does not scale with gift size [Maxwell and Prokop 2018]), as well as an indirect effect on offspring survival (Tuni et al. 2013). There are many possible functions of multimodal signals which should be considered when studying instances of complex courtship. The different signals contained within a bout of multimodal courtship could potentially transfer different pieces of information, transfer redundant information for the sake of signal efficiency or back-up, or achieve purposes besides information transfer (eg, sexual stimulation of the female) (Mitoyen et al. 2019). Additionally, it is possible that some portions of the multimodal signal no longer have an explicit purpose besides increasing the complexity of the display, as more complex signals can be intrinsically more attractive as indicators of male motor performance or neural control (Byers et al. 2010; Mitoyen et al. 2019; Choi et al. 2022). Given that the vibratory component of *P. mirabilis* courtship exhibits limited plasticity in response to gift quality (provided that a nuptial gift is present), it likely serves a simpler function within the multimodal courtship framework. It is possible that this signal serves to capture female attention and direct it toward the nuptial gift, plays a redundant role in communication of male receptivity and/or quality, or transfers information that is ultimately not relevant to mate choice (eg, for purposes of signal or species recognition).

Overall, our findings demonstrate that *P. mirabilis* males do adjust their investment in vibratory courtship signals at a macroscopic level (most notably, they signal for longer when they have a nuptial gift vs. when they do not), though plasticity in vibratory signaling based on nuptial gift quality is slight. While this study contributes to the growing body of work attempting to untangle the several signals which make up the multimodal courtship performed by males of this species, the exact purpose of the characteristic courtship vibrations remains largely unknown. More research into the functional role of this energetically costly signal is needed, as well as a deeper understanding of female preference in *P. mirabilis*, in order to fully interpret sexual investment in this spider.

## Data Availability

Analyses reported in this article can be reproduced using the data provided by Oberweiser et al. (2026).
